# Predicting changes in U.S. county health: What does machine learning tell us about multiple determinants of health

**DOI:** 10.1177/22799036261480261

**Published:** 2026-08-19

**Authors:** Di Yang, David Vanness

**Affiliations:** 1 LSE Health, The London School of Economics and Political Science, London, UK; 2 Department of Health Policy and Administration, The Pennsylvania State University, University Park, USA

**Keywords:** multiple determinants of health, premature mortality, population health, machine learning, XGBoost

## Abstract

**Objective:**

Understanding multiple determinants of health and their contributions to premature mortality is essential for policymaking in population health. As an exploratory forecasting exercise, we aimed to analyze the associations between multiple determinants of health and improvement in premature mortality in U.S. counties.

**Methods:**

Using County Health Rankings data on health determinants from 2010-2014, we predicted changes in premature mortality from 2015 to 2019. We used Extreme Gradient Boosting, which allows for flexible functional estimates of a multi-dimensional health production function, to assess the association between improvement in premature mortality and pre-period health determinants. We made Accumulated Local Effects plots to illustrate these associations and conducted a conventional spatial regression as a robustness check.

**Results:**

We find a negative association between the baseline some-college rate (percentage of adults aged 25-44 with some post-secondary education) and change in premature mortality. A one-percentage-point increase in the baseline some-college rate in 2010-2014 is associated with a reduction of premature mortality by 9.57 years of potential life lost per 100,000 population from 2015 to 2019. The magnitude of the association for each county varied based on each county’s baseline some-college rate.

**Conclusions:**

Our findings reveal that improvements in post-secondary education are associated with relatively large, yet variable, gains in population health across U.S. counties. Applying machine learning methods to estimate a county-level health production function can inform tailored policies to improve local population health.

## Introduction

The U.S. health care system is often considered inefficient and underperforming when compared to other developed industrial nations.^1,2^ Underinvestment in social determinants of health relative to investment in health care may explain this underperformance.^3^ Evidence in the literature suggests that policymakers can improve population health by investing in social services regardless of whether population health improvement is the direct goal.^4^ For example, the Centers for Medicare and Medicaid Services (CMS) launched the Accountable Health Communities initiative, investing $157 million over five years to reduce medical costs by addressing upstream social determinants of health, such as food and housing insecurity.^5^ A CMS case study at Reading Hospital in Pennsylvania showed that avoidable emergency department visits declined by 15% after medical screening and referral were complemented with community services, which highlighted the business value of addressing health-related social needs.^6^

With social investment as a viable approach to improving population health, researchers have long sought benchmarks to predict and monitor potential returns to investment across multiple determinants of health.^7^ Park et al. (2015) used County Health Rankings data to estimate the relative contribution of multiple health determinants to population health.^8^ They grouped individual health determinant measures into four categories: health behaviors, clinical care, social and economic factors, and physical environment. Each category received a composite score based on predetermined weights. Their findings indicated that the “social and economic factors” category had the highest relative contribution to county-level morbidity and mortality. However, contributions for each specific health determinant measure were not reported.

Our research extends the literature on social determinants of health by estimating a county-level health production function, using 2010-2014 multiple determinants of health (baselines and annual rates of change) to predict 2015-2019 change in premature mortality. Specifically, we analyze temporal relationships to estimate changes in future health outcomes as a function of past baselines and annual rates of change in health determinants, rather than levels as a function of levels. We aim to investigate associations between past baselines and annual rates of change in health determinants and future change in premature mortality.

## Methods

We used 2010-2019 County Health Rankings data and excluded data from 2020 onward due to the large, discontinuous effects of the COVID-19 pandemic.^9^ The County Health Rankings project, led by the University of Wisconsin Population Health Institute, gathers health and health determinant data for nearly every county in the United States. Spanning two decades, this dataset offers a comprehensive view of county-level contextual characteristics and their impact on population health. The dataset is publicly available on their website (https://www.countyhealthrankings.org). Although the dataset includes almost all 3,244 U.S. counties, missing data in some measures reduced the analytic sample size to 2,509. We will revisit the missing data issue in the Discussion section.

Our outcome variable is the difference between the 2019 and 2015 levels of premature mortality (hereafter, change in premature mortality), with negative values indicating reductions in premature mortality and improvements in population health. Premature mortality is measured by years of potential life lost (YPLL) per 100,000 population. YPLL is calculated by subtracting the age at death from 75, thereby emphasizing the years lost due to premature mortality.^9^ Therefore, operationally, our outcome variable is the 2019 level of premature mortality (YPLL per 100,000 population) minus the 2015 level of premature mortality (YPLL per 100,000 population) at the county level. County-level YPLL statistics were obtained from the National Center for Health Statistics. Contextual health determinants included 2010-2014 rates of unemployment, uninsurance, child poverty, primary care physicians per 100,000 population, obesity, physical inactivity (percentage of adults reporting no leisure-time physical activity), some-college rate (percentage of adults aged 25-44 with some post-secondary education), and high school graduation rate. The obesity rate may be considered a proxy metric for poor diet,^9^ which may be attributed to community culture and access to fresh produce.^10^ The physical inactivity rate is also a reflection of community culture and lifestyle. To construct predictors from the 2010-2014 health determinants, we estimated county-level baselines and annual rates of change using OLS regressions for each county. We selected this period due to missing data and comparability issues in earlier years. In addition to the County Health Rankings data, we used official 5-year estimates of the poverty rate from the 2010-2014 American Community Survey to compute county-level differences in poverty rates between Black and White residents and between Hispanic and non-Hispanic residents, measuring racial economic disparities.^11^

(1) $$H_{c,t}=a_{c}+b_{c}t+\epsilon_{c,t}$$

To measure baselines and annual rates of change for health determinants, we used a simple OLS regression as equation (1) to compute the baseline (intercept, *a*) and annual rate of change (slope, *b*) for each county. $H_{c,t}$ measured health determinant H in the county c in year *t*. Year *t* (2010-2014) was recoded as 1 to 5, with 2010 as 1 and 2014 as 5. Separate regressions were conducted for each health determinant within each county.

Our empirical analysis estimated a reduced-form temporal health production function f ,

(2) $${\Delta YPLL}_{2019-2015,c}=f\left(a_{2010-2014,c}^{UR},b_{2010-2014,c}^{UR},a_{2010-2014,c}^{CPR},b_{2010-2014,c}^{CPR},\ldots \ldots \right)+\epsilon_{c},$$

where ${\Delta YPLL}_{2019-2015,c}$ is the difference between YPLL level in 2019 and 2015 for county c, and f is an unknown, potentially nonlinear, function of county health determinant baselines a and annual rates of change b , including unemployment rate, uninsured rate (UR), child poverty rate (CPR), primary care physician rate (number of primary care physician per 100,000 population), obesity rate, physical inactivity, some-college rate, and high school graduation rate. We also included the 2010 level of premature mortality as a baseline to control for potential convergence in premature mortality (regression to the mean), as underperforming counties may naturally improve faster.

We used Extreme Gradient Boosting, a machine learning technique based on ensembles of regression trees, which offers advantages over conventional regression models, such as flexibility for potentially nonlinear relationships between predictors and outcomes.^12^ In typical regression analysis, predictors, interaction terms, and functional form (nonlinearities, polynomial orders, etc.) must be assumed *a priori*, with decisions only partially informed by theory. In contrast, predictive analytics using model ensembles aims to generate predictions with minimized out-of-sample prediction error, rather than testing hypotheses with estimated coefficients. Although the resulting model ensemble cannot be easily summarized in an output table, Accumulated Local Effects plots can help assess the strength and shape of functional relationships between predictors and outcomes.^13,14^

The gradient boosting algorithm^15^ begins by fitting a single model (a regression tree) on a bootstrapped sample of the data, excluding some predictors through random feature selection. Unlike the random forest algorithm, which repeats this process with different resampling and predictors, gradient boosting uses the next regression tree to predict the residuals from the first tree. The consensus prediction is a weighted sum of the two trees. In subsequent rounds, each additional regression tree predicts the residual error from the weighted sum of all previous trees. We used cross-validation to tune the model parameters. Further details of the algorithm and tuning are provided in the Methodology Appendix.

We conducted our machine learning analysis in RStudio using XGBoost (version 3.2.1.1) and ParBayesianOptimization (version 1.2.6) to estimate the production function described in equation (2).^16^ We then used the iml package (version 0.11.4) to generate Accumulated Local Effects (ALE) plots. ALE plots can reveal the direction of association and have a clear local interpretation, which is, “conditional on a given value, the *relative effect* of changing the feature on the prediction”.^17,18^ However, we cannot compute an easily interpretable global (overall) effect in real-world terms that is comparable to coefficients in conventional regression-based models. Therefore, we also conducted a conventional spatial regression as a robustness check to confirm the direction of association found in machine learning and report the coefficients from the spatial regression as global effects to aid interpretability. We used a spatial autoregressive model with a generalized spatial two-stage least-squares estimator (spregress in Stata 19), controlling for the spatial lag of neighboring counties. The reporting of this study conforms to the STROBE statement for observational studies [Supplemental File 1].^19^

## Results

**Figure 1.**
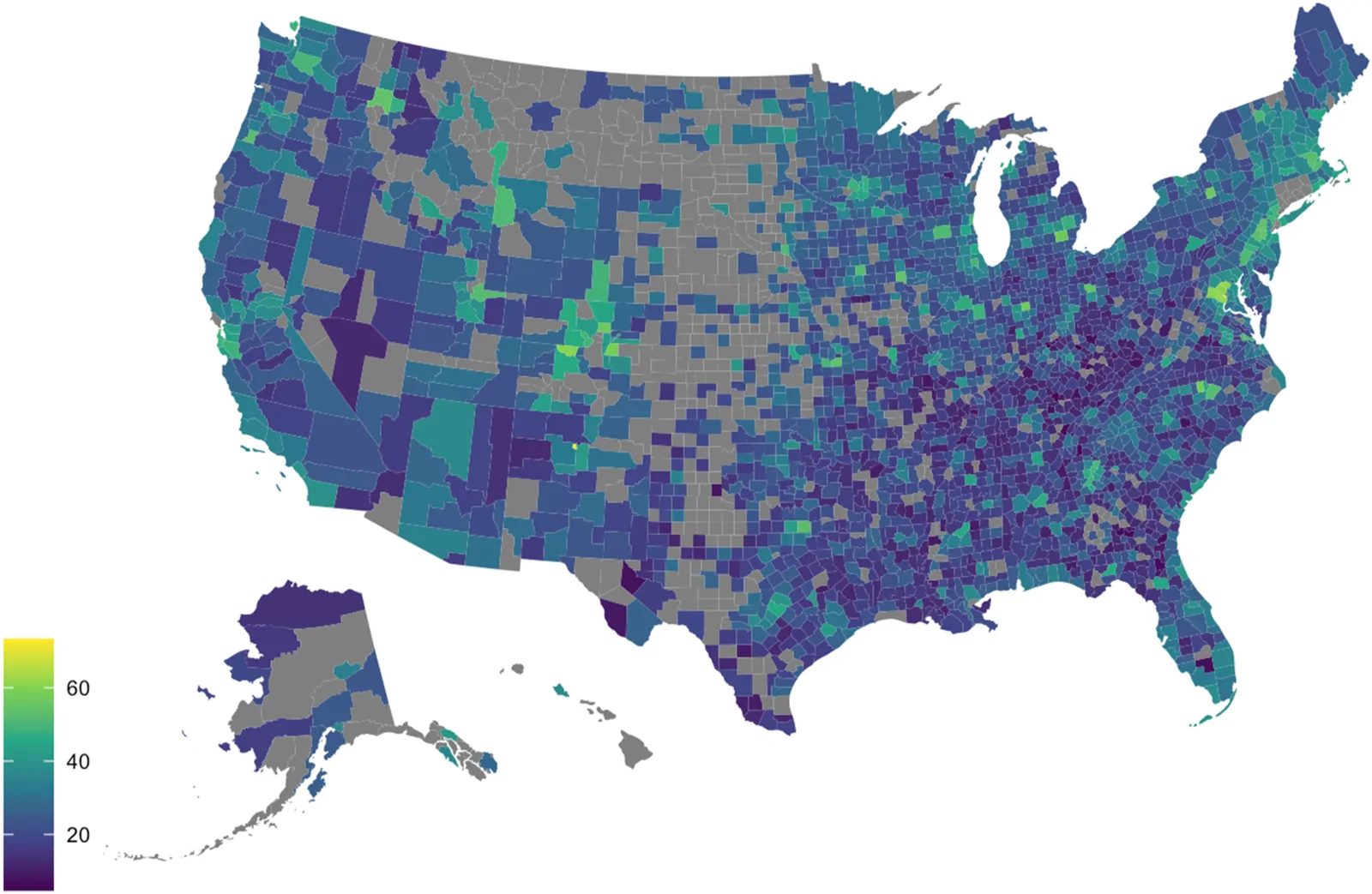
Baseline Some-College Rate in 2010-2014. *Notes*. Some-college rate is the percentage of adults aged 25-44 with some post-secondary education. This map shows the county-level baseline some-college rate in 2010-2014, with brighter colors indicating higher baseline some-college rates. For example, counties colored bright green, corresponding to 60 in the figure legend, suggest that the baseline some-college rate in these counties is 60%.

Table 1 reports descriptive statistics for the outcome and predictor variables. The first row reports our outcome variable, change in premature mortality, and the following rows report the predictors. On average, premature mortality increased by 496 years of potential life lost per 100,000 population from 2015 to 2019, which suggests a worsening overall trend at the county level. The mean annual rates of change for the unemployment rate, uninsured rate, and child poverty rate were negative, reflecting overall improving trends in these socioeconomic health determinants from 2010 to 2014. Conversely, the mean annual rates of change for obesity and physical inactivity rates were positive, suggesting overall declining trends in health culture and lifestyle at the county level. The mean annual rates of change for high school graduation rates and the percentage of adults aged 25-44 with some post-secondary education were also positive, indicating overall improving trends in education at the county level. Meanwhile, we also observe significant regional variation in our predictors. For example, Figure 1 shows a map of U.S. counties colored by the baseline some-college rate between 2010 and 2014, with brighter colors indicating higher baseline some-college rates. We observe significant regional variation on the map. For instance, many counties in the Northeastern states are colored bright green, showing a high baseline some-college rate of around 60%. In contrast, many counties in the Central Appalachia region are colored dark purple, suggesting a baseline some-college rate below 20%. We will revisit this regional variation in the Discussion section and connect it with our main results.

**Table 1.** Descriptive statistics.

​	​	Mean	Minimum	Maximum	Standard deviation
Outcome variable (2019 - 2015 difference)					
Years of potential life lost (YPLL) rate per 100,000 population	​	495.84	-4693.06	10097.34	1106.10
Predictor Variables (2010-2014)					
Unemployment rate: percent of population age 16+ unemployed and looking for work	Baseline	10.65	1.80	31.00	3.20
	Annual Rate of Change	-0.81	-3.30	1.10	0.41
Uninsured rate: percent of population < 65 without insurance	Baseline	19.37	3.80	40.30	5.54
	Annual Rate of Change	-0.86	-2.50	0.50	0.45
Child poverty rate: percent of children (under age 18) living in poverty	Baseline	24.71	2.80	59.80	8.86
	Annual Rate of Change	-0.07	-4.20	3.00	0.66
Primary care physicians (PCP) rate: (Number of PCP/population) * 100,000	Baseline	64.67	-12.52	721.64	47.41
	Annual Rate of Change	-1.82	-97.09	29.07	5.78
Obesity rate: percent of adults that report BMI >= 30	Baseline	28.06	12.10	42.53	3.72
	Annual Rate of Change	0.58	-1.97	3.01	0.61
Physical inactivity: percent of adults that report no leisure time physical activity	Baseline	26.85	9.24	48.30	5.57
	Annual Rate of Change	0.18	-3.16	3.98	0.78
Some college: percent of adults aged 25-44 with some post-secondary education	Baseline	23.68	4.69	73.30	9.29
	Annual Rate of Change	7.56	1.57	12.49	1.53
High school graduation rate	Baseline	76.37	20.21	114.74	12.97
	Annual Rate of Change	1.23	-16.81	16.61	2.76
Poverty rate difference between black and white residents	​	17.94	-29.90	92.30	18.83
Poverty rate difference between Hispanic and Non-Hispanic residents	​	15.23	-35.60	90.10	14.56

N = 2,509.

*Notes.* Baseline (intercept) and annual rate of change (slope) are from the linear regression in which year is the independent variable.

Table 2 reports the feature (predictor) importance in our tree-based model. Gain is the average improvement in the loss function, squared error in our case, when making a split. Gain is a percentage contribution, and a higher percentage means higher importance.^16^ This is different from the p-value or the coefficient in conventional regression models. Gain measures the importance of a feature in the process of fitting the tree-based model. It does not have a practical interpretation of the association between the predictor and outcome, nor does it measure whether the association is statistically significant. Table 2 shows that the obesity rate (baseline), some-college rate (baseline), and physical inactivity rate (baseline) are the top predictors for feature importance. The 2010 level of premature mortality ranks second in feature importance, which suggests the importance of “regression to the mean” in fitting our tree-based model. However, as cautioned above, the associations between these predictors and the outcome are not necessarily statistically significant, nor are they the largest in the magnitude of association, as feature importance is only a metric for the process of fitting our tree-based model.

**Table 2.** Feature importance.

Feature	Gain
Obesity rate (baseline)	0.088
Premature mortality (2010 level)	0.080
Some-college rate (baseline)	0.077
Physical inactivity rate (baseline)	0.075
Uninsured rate (baseline)	0.066
High school graduation rate (annual rate of change)	0.061
Unemployment rate (baseline)	0.060
Physical inactivity rate (annual rate of change)	0.054
Child poverty rate (baseline)	0.054
Primary care physician rate (annual rate of change)	0.048
High school graduation rate (baseline)	0.048
Child poverty rate (annual rate of change)	0.048
Some-college rate (annual rate of change)	0.045
Poverty rate difference between black and white residents	0.041
Poverty rate difference between Hispanic and Non-Hispanic residents	0.039
Obesity rate (annual rate of change)	0.038
Primary care physician rate (baseline)	0.034
Uninsured rate (annual rate of change)	0.027
Unemployment rate (annual rate of change)	0.016

*Notes.* This table reports fractional contribution of each feature (predictor) to the model based on the total gain of this feature’s splits, which is a technical metric and does not have practical interpretation on the association between the feature and outcome. Higher percentage means higher importance. Premature mortality (2010 level) is included as baseline to control for regression to the mean.

**Figure 2.**
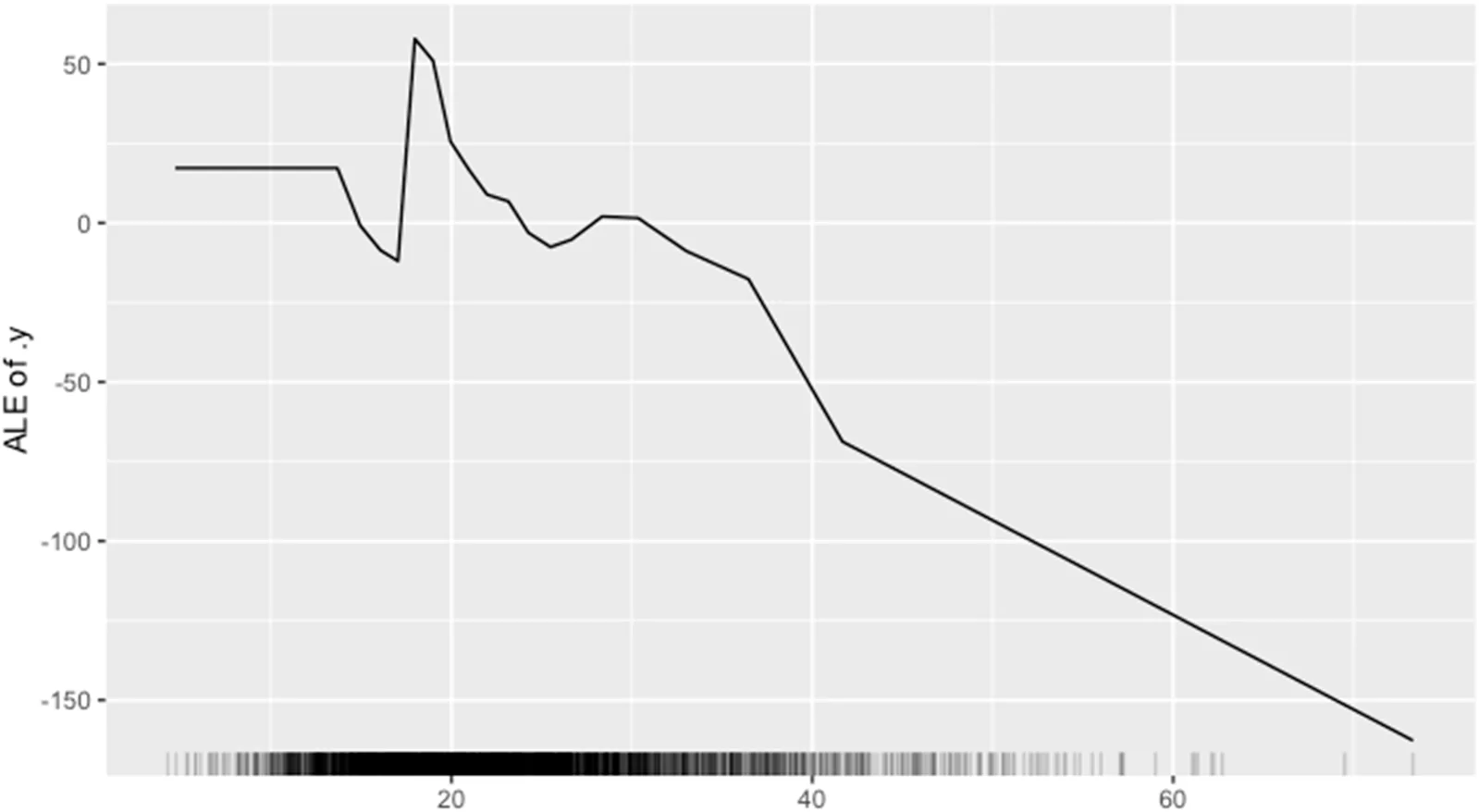
Accumulated Local Effects Plot for Baseline Some-College Rate. *Notes*. Some-college rate is the percentage of adults aged 25-44 with some post-secondary education. This figure shows the accumulated local effect of the baseline some-college rate across its value range. The x-axis is the baseline some-college rate, and the y-axis is the accumulated local effects on the outcome. It can imply the overall direction of association between baseline some-college rate and change in premature mortality, but cannot be interpreted as marginal effects or coefficients in a conventional regression model.

**Appendix Figure 1.**
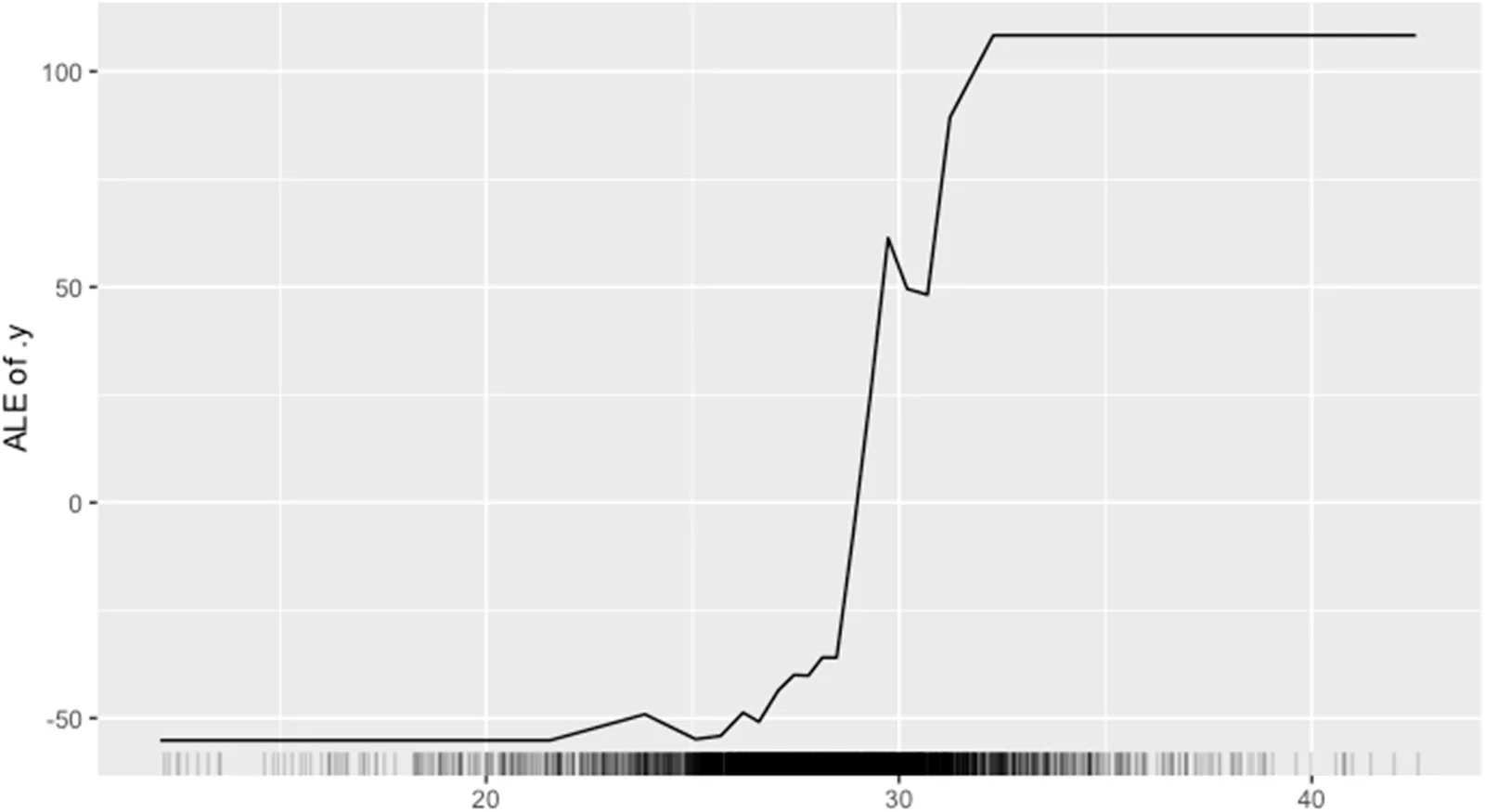
Accumulated Local Effects Plot for Baseline Obesity Rate. *Notes.* Obesity rate is the percentage of adults reporting a BMI of 30 or higher. This figure shows the accumulated local effect of the baseline obesity rate across its value range. The x-axis is the baseline obesity rate, and the y-axis is the accumulated local effects on the outcome. It can imply the overall direction of association between baseline obesity rate and change in premature mortality, but cannot be interpreted as marginal effects or coefficients in a conventional regression model.

**Appendix Figure 2.**
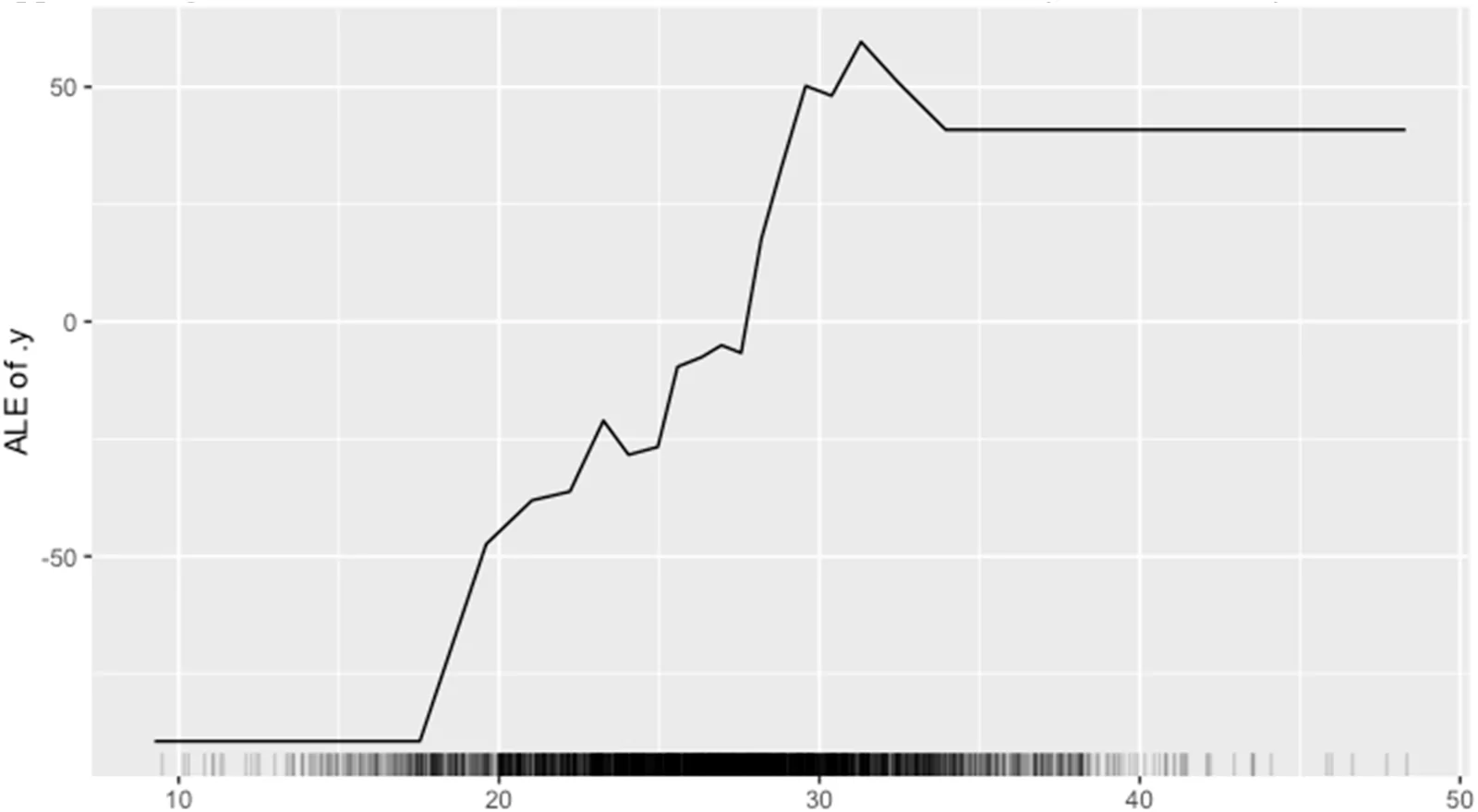
Accumulated Local Effects Plot for Baseline Physical Inactivity Rate. *Notes*. Physical inactivity rate is the percentage of adults reporting no leisure-time physical activity. This figure shows the accumulated local effect of the baseline physical inactivity rate across its value range. The x-axis is the baseline physical inactivity rate, and the y-axis is the accumulated local effects on the outcome. It can imply the overall direction of association between baseline physical inactivity rate and change in premature mortality, but cannot be interpreted as marginal effects or coefficients in a conventional regression model.

To facilitate interpretation of the associations in real-world terms, we generated Accumulated Local Effects plots for the top predictors. Figure 2 is the ALE plot for baseline some-college rate and suggests a negative association between the baseline some-college rate in 2010-2014 and the change in premature mortality in 2015-2019. In other words, a higher baseline some-college rate is associated with a larger reduction in premature mortality. Appendix Figure 1 is the ALE plot for the baseline obesity rate and suggests a positive association between the baseline obesity rate in 2010-2014 and the change in premature mortality in 2015-2019. In other words, a higher baseline obesity rate is associated with a larger increase in premature mortality. Appendix Figure 2 is the ALE plot for the baseline physical inactivity rate and suggests a positive association between the baseline physical inactivity rate in 2010-2014 and the change in premature mortality in 2015-2019. In other words, a higher baseline physical inactivity rate is associated with a larger increase in premature mortality.

Table 3 reports the results from our robustness check using spatial regression. The coefficients for baseline some-college rate, baseline obesity rate, and baseline physical inactivity rate confirm the direction of association found in the ALE plots above. However, only the coefficient for the baseline some-college rate is statistically significant. A one-percentage-point increase in the baseline some-college rate in 2010-2014 is associated with a reduction of premature mortality by 9.57 YPLL per 100,000 population from 2015 to 2019. Furthermore, we found a statistically significant association between the annual rate of change in child poverty rate and the change in premature mortality, although this predictor ranked relatively low in feature importance. A one-percentage-point increase in the child poverty rate per year in 2010-2014 is associated with an increase of premature mortality by 118.55 YPLL per 100,000 population from 2015 to 2019.

**Table 3.** Robustness Check using Spatial Regression.

​	Change in premature mortality
Unemployment rate (baseline)	18.01
Unemployment rate (annual rate of change)	34.09
Uninsured rate (baseline)	-7.34
Uninsured rate (annual rate of change)	16.89
Child poverty rate (baseline)	8.36
Child poverty rate (annual rate of change)	118.55***
Primary care physician rate (baseline)	0.50
Primary care physician rate (annual rate of change)	-2.95
Obesity rate (baseline)	8.44
Obesity rate (annual rate of change)	54.07
Physical inactivity rate (baseline)	0.25
Physical inactivity rate (annual rate of change)	-17.39
Some-college rate (baseline)	-9.57**
Some-college rate (annual rate of change)	-11.22
High school graduation rate (baseline)	-1.11
High school graduation rate (annual rate of change)	-11.67
Poverty rate difference between black and white residents	-0.64
Poverty rate difference between Hispanic and Non-Hispanic residents	0.13
Premature mortality (2010 level)	-0.03

*Notes.* This table reports coefficients from a conventional spatial regression. The outcome variable is the 2019 level of premature mortality (YPLL per 100,000 population) minus the 2015 level of premature mortality (YPLL per 100,000 population) at the county level. * p<0.1, ** p<0.05, *** p<0.01.

## Discussion

As an exploratory forecasting exercise, our contribution is in the flexible machine learning approach to estimate county-level population health production functions. Our study used gradient boosting to estimate associations between past baselines and annual rates of change in health determinants and future change in premature mortality. This machine learning approach accommodates nonlinearity in the population health production function, highlighting the variation in associations between health determinants and population health outcomes across counties. This is particularly useful for tailored county-level policymaking, as different counties may be at different levels of health determinants, leading to varying associations between health determinants and population health.

Baseline some-college rate, baseline obesity rate, and baseline physical inactivity rate were identified as the three factors with the highest feature importance in our tree-based model. Notably, all top factors are 2010-2014 baselines, rather than the annual rates of change, of the health determinants. Our robustness check using conventional spatial regression also confirms a statistically significant association between baseline some-college rate in 2010-2014 and change in premature mortality from 2015 to 2019. A one-percentage-point increase in the baseline some-college rate in 2010-2014 is associated with a reduction of premature mortality by 9.57 YPLL per 100,000 population from 2015 to 2019. We also observe notable variations in the association across the value range of a predictor, as shown in the varying slope in ALE plots across the value range of a predictor.^17,18^ This is particularly important as health determinants can vary widely across counties, for example, baseline some-college rate ranging from around 60% in the Northeastern states to below 20% in the Central Appalachia region (Figure 1).

Our study has important limitations. First, our analysis only shows the association between baseline some-college rate and changes in premature mortality; it does not establish causality. “Prediction” in our study is used in the technical sense in machine learning models and does not imply causality. Moreover, our study is constrained by data limitations. Data quality and comparability issues limit our choice of health outcome to premature mortality. We did not consider other potentially important health outcome measures in the County Health Rankings, such as the average number of reported mentally unhealthy days per month, physically unhealthy days per month, and the percentage of adults reporting fair or poor health, because their values were imputed rather than directly reported by the respondents, raising concerns about data quality. Furthermore, we excluded data from 2020 onward due to the significant and discontinuous effects of the COVID-19 pandemic. COVID-19 was the third leading cause of death in 2020 and 2021, contributing to a decline in U.S. life expectancy.^20,21^ However, its impact on mortality has been decreasing since 2022, ranking as the fourth leading cause of death in 2022 and the tenth in 2023.^22,23^ The pandemic could also confound health determinants (i.e., physical inactivity) and premature mortality substantially during that timeframe, so we excluded this period from our analysis. Unfortunately, due to the temporal nature of our approach requiring long-term longitudinal data, rerunning the analysis in a “post-COVID” era could not be accomplished for several years.

**Figure 3.**
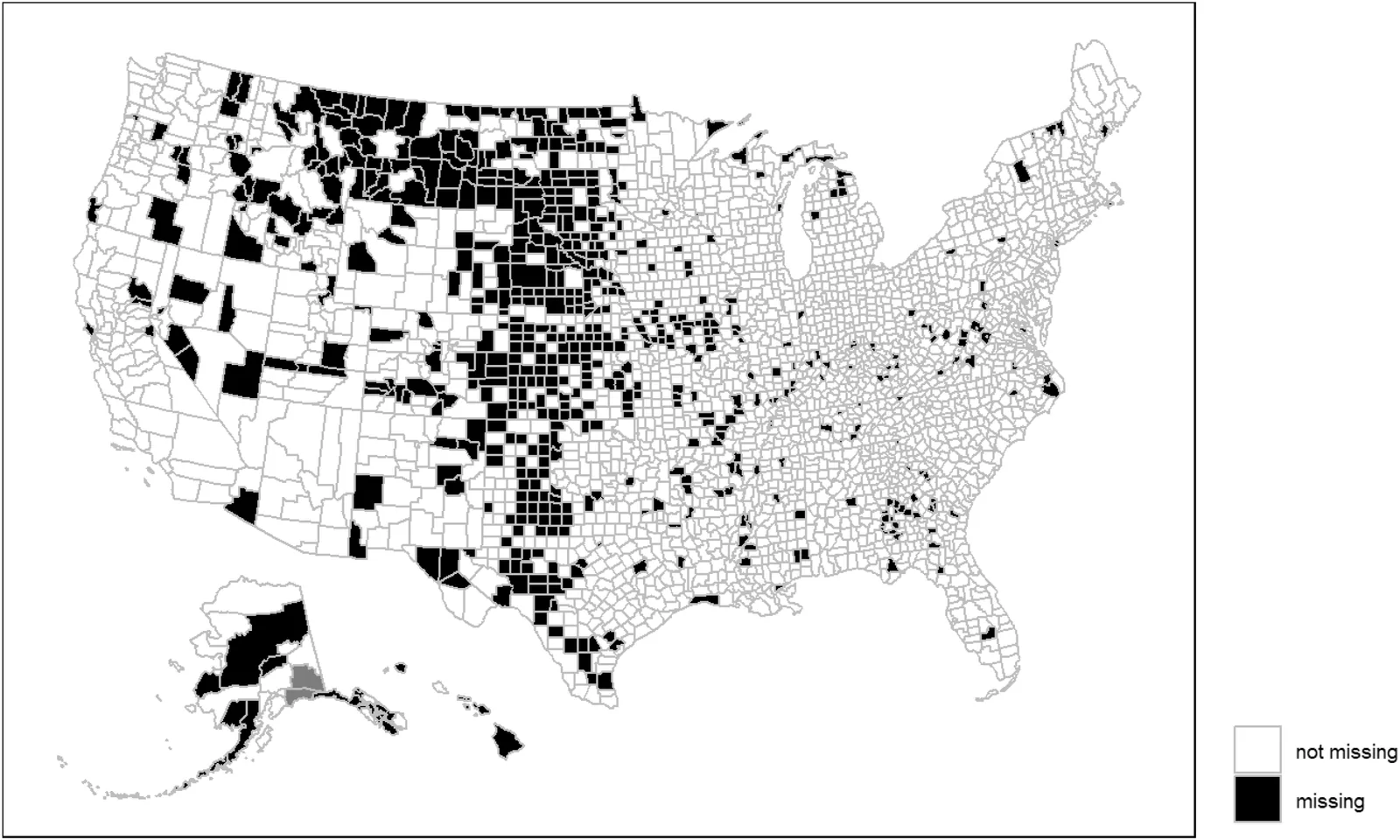
Map of U.S. Counties with Missing Data. *Notes*. This figure shows a map of US counties. The counties with missing data are colored black.

Furthermore, the missing data issue may constrain the generalizability of our findings. Our analytic sample includes 80% of all U.S. counties. As shown in Figure 3, the counties with missing data were mostly in the West North Central and Mountain Census Divisions, which would limit the generalizability of our findings in these regions. We did not perform imputation for missing counties because they are very geographically concentrated. This clustering of missing counties (e.g., Mountain West and Northern Rockies) suggests that they are likely systematically different from non-missing counties (e.g., Northeastern states) in unobserved characteristics, violating the assumption of imputation.

## Conclusion

Using a flexible machine learning approach and County Health Rankings data, we find that improvements in post-secondary education are associated with relatively large, yet variable, gains in population health across U.S. counties. Moreover, our study provides a cautionary tale against “one-size-fits-all” in population health policymaking, as the association between health determinants and population health outcomes may vary based on the county’s level of health determinants. Meanwhile, our study also points to potential limitations of applying machine learning methods to real-world policymaking in population health. Unlike coefficients in a conventional regression model, our tree-based model and ALE plots do not provide a straightforward and easily interpretable overall effect in real-world terms. Future methodological research should therefore focus on real-world interpretability to translate complex tree-based outputs into actionable, population-level effect estimates for public health decision-making.

## Supplemental material

Supplemental material - Predicting changes in U.S. County Health: What does machine learning tell us about multiple determinants of health Supplemental material for Predicting changes in U.S. county health: What does machine learning tell us about multiple determinants of health by Di Yang and David Vanness in Journal of Public Health Research.

Supplemental material - Predicting changes in U.S. County Health: What does machine learning tell us about multiple determinants of health Supplemental material for Predicting changes in U.S. county health: What does machine learning tell us about multiple determinants of health by Di Yang and David Vanness in Journal of Public Health Research.

Supplemental material - Predicting changes in U.S. County Health: What does machine learning tell us about multiple determinants of health Supplemental material for Predicting changes in U.S. county health: What does machine learning tell us about multiple determinants of health by Di Yang and David Vanness in Journal of Public Health Research.

## Data Availability

The dataset is publicly available from the website of the County Health Rankings (https://www.countyhealthrankings.org).
