# Supplementary material for: Predicting changes in U.S. county health: What does machine learning tell us about multiple determinants of health
Source: J Public Health Res. 2026 Aug 19;15(3):22799036261480261. doi: 10.1177/22799036261480261 (PMC13490815; doi:10.1177/22799036261480261)

Methodology Appendix

Greedy Algorithm

Appendix Figure A is a demonstration of a "greedy" algorithm to fit a single recursive partition (regression tree). Beginning with the entire dataset, the algorithm searches for a predictor x that splits the sample into two segments, R_1 and R_2, at the cutting point of x=s. The mean response in each segment, $\bar{y}_{R1}$ and $\bar{y}_{R2}$, is the predicted outcome for all observations in that partition and the Residual Sum of Squares (RSS) can be computed accordingly. The choice of splitting predictor x and cut point s is determined by minimizing RSS as shown in the formula below. The splitting process is repeated (recursively) on each partition until a preset criterion is met, such as a minimum number of observations in a partition. "Greedy" in this illustration refers to only making the greatest reduction in RSS at each split, potentially disregarding splits that would allow for more productive splits further down in the tree.[1]

$$RSS = \sum_{j=1}^{J} \sum_{i \in R_j} (y_i - \overline{y_{R_j}})^2$$

The above process involves the construction of a single recursive partition. Typically, single tree models are fairly weak in their ability to predict outcomes. Bagging (bootstrapped aggregation) combines the predictions from multiple recursive partitions constructed on different resampled datasets, allowing for discovery of relationships in subsets of data often not found in a single tree constructed using a greedy algorithm. Furthermore, each individual tree has high variance because of its dependence on the resampled dataset. Averaging these trees can reduce the variance. Similarly, random feature selection constructs trees using only a subset of predictor variables. By excluding strong predictors, greedy tree construction

algorithms can be nudged into finding relationships that may have been masked by strong predictors defining splits at the top of the tree. Ensembles of relatively weak models using methods such as random forest and gradient boosting can often outperform single strong regression models in terms of out-of-sample prediction error.[2,3]

Boosting Algorithm

Generally, the boosting algorithm proceeds as follows:[1]

a. Set $r_i = y_i$ for all observations in the training set. r_i represent the residual for observation *i*.
b. Fit a tree $\hat{f}(x)$ with *s* splits to predict r_i
c. Update the residual r_i by subtracting the tree prediction with a shrinkage parameter $r_i \leftarrow r_i - \lambda\hat{f}(x)$
d. Repeat step b and c for a pre-set number of times *n*.

Building on the example in **Appendix Figure A**, in each terminal segment, the difference between ȳ and the actual outcome y is the residual for each observation. These residuals will be used as the new y to fit the next tree. Hence, boosting fits a sequence of trees, and each tree is fitted on the residual of the previously fitted tree, as if the algorithm is "learning" from the previous errors. The final result would be the weighted summation of all trees.

Tuning Model Parameters

Because both bagging and random feature selection use randomization as part of the estimation process, it is important to repeat the algorithm multiple times using different random seed values. We use 10-time repeated 10-fold cross-validation to tune the model parameters and minimize Root-Mean-Square Error (RMSE). We randomly divide the sample

into 10 equal-sized subsets ("folds"), 9 of which are used to estimate the ensemble of models, then cross-validation RMSE is calculated in the 1 remaining fold that was not used for estimation. The process is repeated such that each fold is used for estimation 9 times and for out-of-sample validation once. Then, we calculate the average cross-validation RMSE across 10 folds for this round of repetition. The entire 10-fold cross-validation process is then repeated 10 times. Finally, we average the RMSE from 10 repetitions for the final RMSE.

RMSE is determined in part by several "tunable" parameters in the boosting algorithm – including the number of trees fitted, the number of splits in each tree, and the shrinkage parameter. The number of trees fitted is n and a large n can overfit the model. The complexity of the boosted ensemble is controlled by s, the number of splits in each tree. The shrinkage parameter λ controls the rate of learning by applying declining weights to subsequent trees in the sequence. Further optimization of the boosting model involves stochastic resampling of data and subsampling of predictors. We can bootstrap the training set to get a $p\%$ sample and run the boosting algorithm. Moreover, at each split, only a random $q\%$ subsample of all predictors is used to make the split so that predictions from different trees are decorrelated. The size of the bootstrapped sample p and the size of predictor subsample q also need to be tuned to minimize RMSE.[1]

Reference

1. James G, Witten D, Hastie T, Tibshirani R. *An Introduction to Statistical Learning with Applications in R*. Vol 112. Springer; 2013.
2. Dietterich TG. Ensemble methods in machine learning. doi:10.1007/3-540-45014-9_1
3. Friedman JH. Greedy function approximation: A gradient boosting machine. *The Annals of Statistics*. 2001;29(5):1189-1232. doi:10.1214/aos/1013203451

Appendix Figure A. "Greedy" Illustration of Fitting a Tree

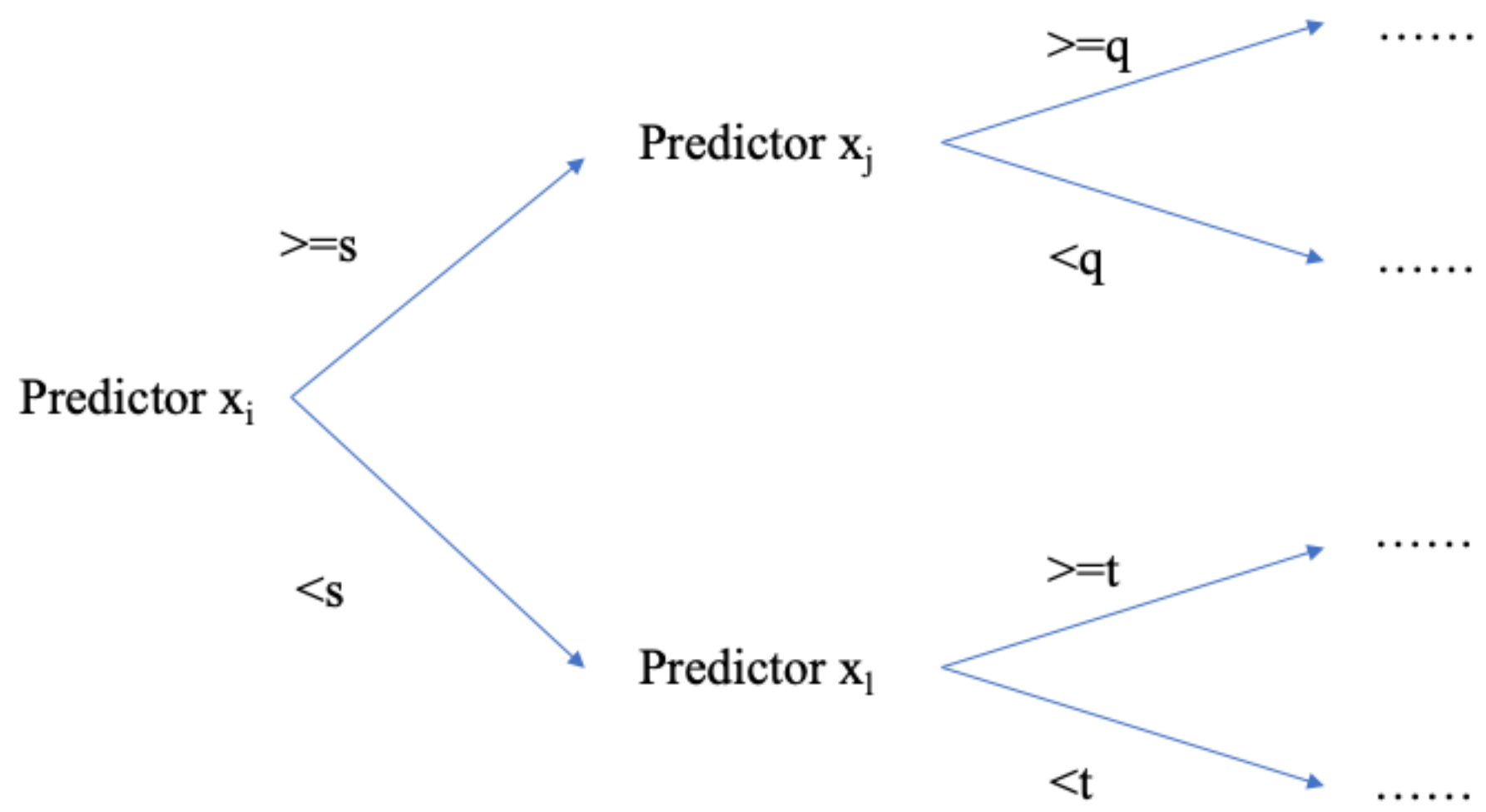

Supplement: Supplementary file 1 [file sj-pdf-1-phj-10.1177_22799036261480261.pdf]
