# Supplementary material for: Predicting changes in U.S. county health: What does machine learning tell us about multiple determinants of health
Source: J Public Health Res. 2026 Aug 19;15(3):22799036261480261. doi: 10.1177/22799036261480261 (PMC13490815; doi:10.1177/22799036261480261)

R Code for the Maps

```
## -- Attaching core tidyverse packages ------------------------ tidyverse 2.0.0 --
## v dplyr     1.2.1     v readr     2.2.0
## v forcats   1.0.1     v stringr   1.6.0
## v ggplot2   4.0.3     v tibble    3.3.1
## v lubridate 1.9.5     v tidyr     1.3.2
## v purrr     1.2.2
## -- Conflicts ------------------------------------------ tidyverse_conflicts() --
## x dplyr::filter() masks stats::filter()
## x dplyr::lag()    masks stats::lag()
## i Use the conflicted package (<http://conflicted.r-lib.org/>) to force all conflicts to become errors
```

```r
#map of missing counties
load(file = "analyticwrace.Rdata")
analyticwrace$missing=0
formap=analyticwrace %>% select(FIPS,missing)

all_counties = usmap::countypop %>% select(fips) %>% mutate(fips = as.character(fips))

missing_map = all_counties %>%
  left_join(formap %>% rename(fips = FIPS), by = "fips") %>%
  mutate(missing = if_else(is.na(missing), 1, 0),
         miss_f = as.character(missing))

# plot: black = missing, white = present
plot_usmap(regions = "counties", data = missing_map, values = "miss_f", color = "grey") +
  theme(panel.background = element_rect(colour = "black")) +
  scale_fill_manual(values = c(`0` = "white", `1` = "black"),
                    name = "",
                    labels = c("not missing", "missing")) +
  theme(legend.position = "right")
```

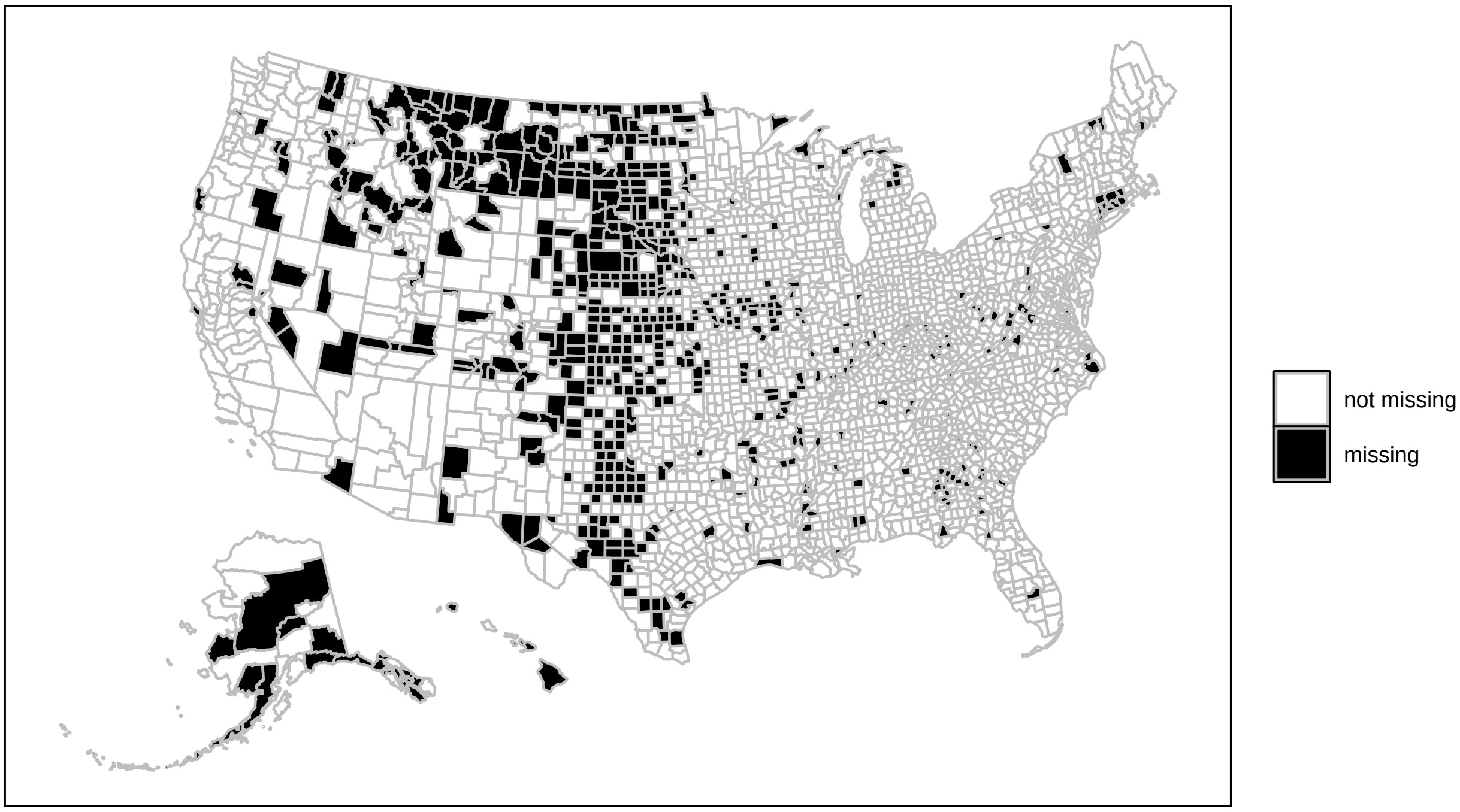


```r
# map of some college rate (baseline)
analyticwrace$fips <- analyticwrace$FIPS
plot_usmap(regions = "counties", data = analyticwrace, values = "somecollegea", color = NA) +
  scale_fill_viridis_c(option = "viridis", name = "")
```

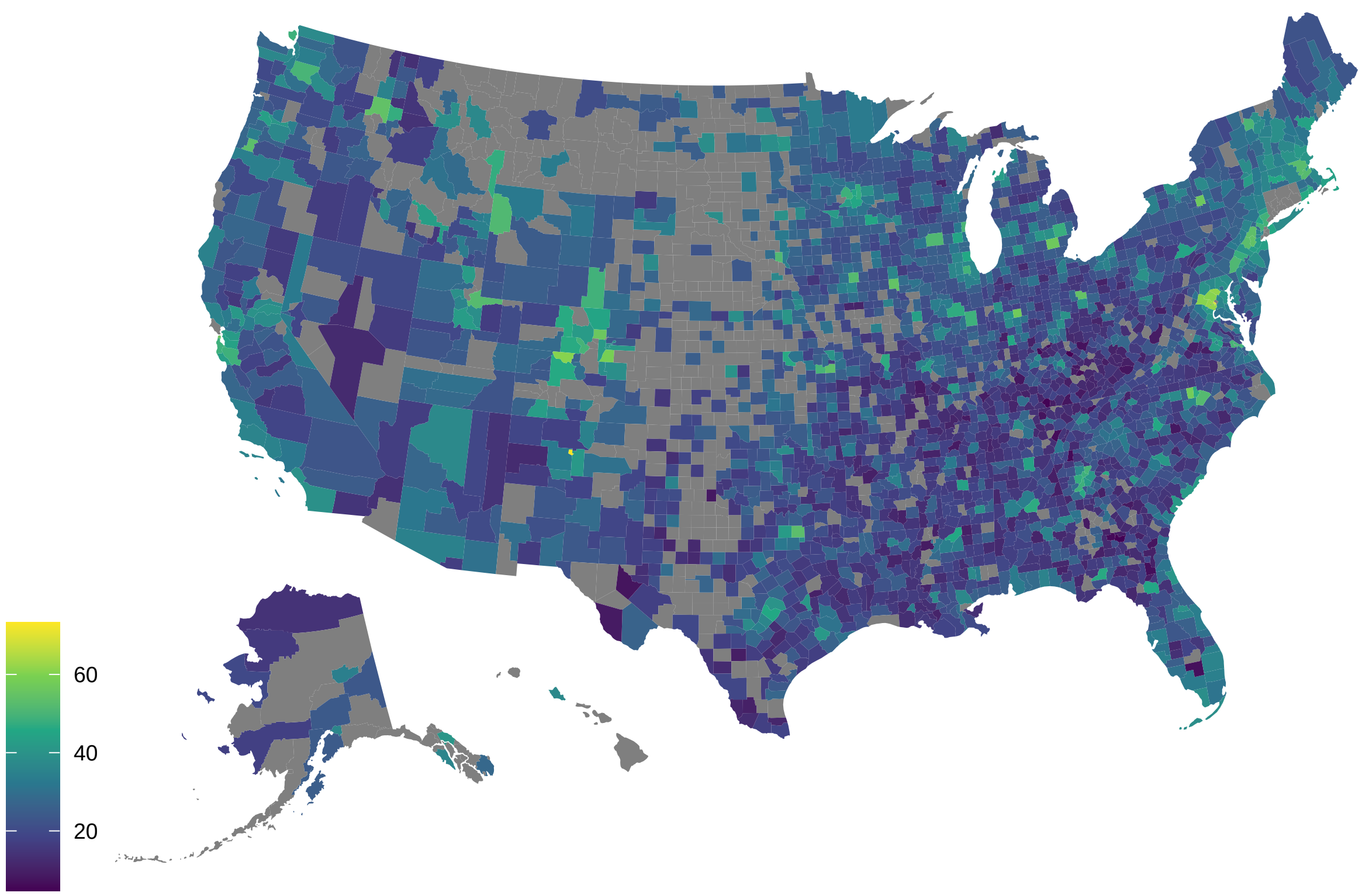

Supplement: Supplementary file 2 [file sj-pdf-2-phj-10.1177_22799036261480261.pdf]
